# Fruit economic characteristics and yields of 40 superior *Camellia oleifera* Abel plants in the low-hot valley area of Guizhou Province, China

**DOI:** 10.1038/s41598-022-10620-2

**Published:** 2022-04-29

**Authors:** Lu Yang, Chao Gao, Jiajun Xie, Jie Qiu, Quanen Deng, Yunchao Zhou, Desheng Liao, Chaoyi Deng

**Affiliations:** 1 Institute for Forest Resources and Environment of Guizhou, Key Laboratory of Forest Cultivation in Plateau Mountain of Guizhou Province, College of Forestry, Guizhou University, Jiaxiu South Road, Guiyang, 550025 China; 2 Guizhou Southwest Karst Regional Development Institute, Xingyi, 562400 China

**Keywords:** Plant sciences, Plant breeding

## Abstract

In this study, we assessed 26 economic characteristics and yields of the mature fruit of 40 superior *Camellia oleifera* Abel plants grown at the *C. oleifera* germplasm resource nursery in the low-hot valley area of Southwest Zuizhou, China, using principal component analysis (PCA). Correlations among the characteristics and the variability of the plants in these characteristics were also analyzed. Out of the 26 characteristics, 16 primary economic characteristics were selected for comprehensive assessment, based on the results of which the plants were ordered to obtain excellent *C. oleifera* germplasms. The data were subjected to PCA, and the 16 characteristics were integrated into 6 independent comprehensive indices, which included PV1 (single-fruit weight), PV2 (pericarp thickness), PV3 (seed rate), PV4 (total unsaturated fatty acids), PV5 (iodine value) and PV6 (dry seed rate). Then, the sum of the products of the contribution rates of the components and components scores was taken as the comprehensive score of each superior plant. In *C. oleifera* grown in the low-hot valley area, the oil yield exhibited very significant positive correlations with the dry seed rate and kernel rate but a very significant negative correlation with the 100-seed weight. The dry seed rate exhibited very significant negative correlations with the fruit diameter and fresh seed rate. Among the 26 characteristics, the variations of the acid value, peroxide value, number of fertile seeds, 100-seed weight and single-fruit weight were great; those of the fruit diameter, fruit height, kernel yield, oleic acid and total unsaturated fatty acid were small, showing strong genetic stability. According to the obtained comprehensive scores, the top 10 plants were ordered as follows: CY-6 > CY-13 > CY-31 > CY-11 > CY-16 > CY-22 > CY-28 > CY-23 > CY-24 > CY-29. This result was basically consistent with the ranking result according to the average yield per unit crown width within five years. In the low-hot valley area of Guizhou, *C. oleifera* exhibits excellent performance in single-fruit weight, total unsaturated fatty acids and kernel rate, 6 characteristics, i.e., acid value, peroxide value, single-fruit weight, the number of fertile seeds, 100-seed weight and α-linolenic acid possess high breeding potentials.

## Introduction

*Camellia oleifera*, belonging to the genus of *Camellia* of the Theaceae family, is a woody oil tree species that is endemic to China. It has gained a fame of one of the four important woody oil plants across the world (the other three are *Olea europaea*, *Elaeis guineensis* and *Cocos nucifera*). Because *C. oleifera* oil contains a fatty acid content comparable to that of olive oil, it is also known as “oriental olive oil”. China is the largest *C. oleifera* oil production country in the world^1^. In recent years, with the improvement of Chinese living standards, Chinese people's demand for edible vegetable oil has been increasing. China has become a large consumer and importer of edible vegetable oil, with an import dependence of approximately 70%. To improve this situation, China has vigorously developed woody oil crops, such as *C. oleifera*. The low-hot valley area of Guizhou Province in China is one of the main distribution areas of *C. oleifera* in China, with a history of more than 300 years. *Camellia oleifera* production in this area plays an important role in the development of woody oil plants in Southwest China.

The low-hot valley areas in Guizhou Province, China, are mainly located in Ceheng and Wangmo Counties in Southwest Guizhou. In these areas, *Camellia oleifera* Abel forest is widely distributed; these areas are also the main distribution areas of *C. oleifera* in Guizhou Province. There, *C. oleifera* has been planted for more than 300 years, and Ceheng County has even won the title of “The famous county of Chinese oil tea”. The growth of *C. oleifera* in the low and hot valley areas has an extremely important strategic significance for the development of woody oil in Guizhou Province. Although these areas own unique conditions for the growth of *C. oleifera*, low-quality and–efficiency bottleneck problems such as cultivar hybridization and low fruit and oil yields have long been existing, which seriously restrict the development of local *C. oleifera* industry. In recent years, scientific and technological workers have carried out research on how to increase the yield of *C. oleifera* from a variety of aspects, such as fertilization measures^2^, site quality^3^, production and management mode of the *C. oleifera* forest^4^ and configuration of pollination trees^5^, which has deepened the understanding of the breeding methods of superior *C. oleifera* cultivars. Also, scholars have reached a consensus that to make full use of the local germplasm resources, based on which to screen and cultivate superior cultivars with powerful adaptability, is the fastest, most economical and most effective way to breed *C. oleifera*^6–10^.

Comprehensive assessment of economic characteristics is an important step of economic forest species breeding. For a long time, the assessment of excellent germplasms of *C. oleifera* have relied on single indexes such as yield and oil content, and there has been a lack of systematic and comprehensive evaluation system for the fatty acid composition and physicochemical properties of *C. oleifera*. Nowadays, the frequently-adopted methods for economic characteristic assessment include grey correlation analysis, technique for order preference by similarity to ideal solution (TOPSIS), rationalization-satisfaction and fuzzy comprehension evaluation. However, all these methods require an ideal value artificially constructed for each index, and therefore, are somewhat subjective. Principal component analysis (PCA) is a multivariate statistical method which is commonly used in comprehensive evaluation and screening in recent years; it achieves the goal of data simplification by reducing dimensions. Comprehensive assessment often involves multiple variables and a large amount of information. To simplify complex data, PCA removes the related and repeated information among multiple variables while retaining as much irrelevant information as possible. Then, the remaining information is integrated, which constitutes the principal components. The simplified principal components are not just simple but contain most of the original information as well^11^. PCA cannot only be used for comprehensive assessment but also for the discovery of potential excellent germplasms via variability and correlation analyses. To date, PCA has been applied in the comprehensive assessment and superior germplasm screening of Yuping *C. oleifera*^12^, Luchuan *C. oleifera*^13^, Ya’an *C. oleifera*^14^, Yunnan Dehong *C. oleifera*^15^ and Jiangxi *C. oleifera*^16^ It has also been used for assessing the influence of growth-promoting bacteria on the yield and quality indexes of organic agricultural tomatoes^17^, analyzing the 13 character indexes of tomatoes and the salt tolerance of 6 tomato germplasm resources^18^ and comprehensively assessing the 12 characteristic indexes of superior grain plants^19^. In addition, PCA has also been applied in the comprehensive evaluation of rice^20,21^, sweet cherries^22^, walnuts^23^, corn^24^, pomegranates^25^, cowpeas^26^ and broad beans^27^.

Based on a previous investigation of *C. oleifera* resources in the low-hot valley of Southwest Guizhou, our team collected 395 early fruiting and high-yield germplasm resources. Out of these resources, we further selected 40 disease-resistant plants with a high- and -stable yield as the candidates of fine breeding. In this study, using the 40 superior *C. oleifera* trees growing in the resource nursery (constructed by our team) as the study objects, we performed descriptive statistical analysis and correlation analysis of the 26 economic characteristics of the mature fruit of the plants. In addition, we performed PCA upon the 16 primary economic characteristics, including single-fruit weight, fruit diameter, fruit height, pericarp thickness, number of fertile seeds, 100-seed weight, oil yield, fresh seed rate, dry seed rate, kernel yield, total unsaturated fatty acids, polyunsaturated fatty acids, acid value, iodine value, saponification value and peroxide value, to investigate the correlation and variation degree among the characteristics of *C. oleifera* in the low-hot valley of Guizhou Province, discover the potential excellent characteristics of the plant in this region, and screen out the single plant with satisfactory comprehensive scores. The results of this study may provide material guarantee and theoretical basis for the breeding and germplasm Innovation of *C. oleifera* in low-heat valley areas.

## Results

### Characteristic correlation testing and analysis

**Figure 1:**
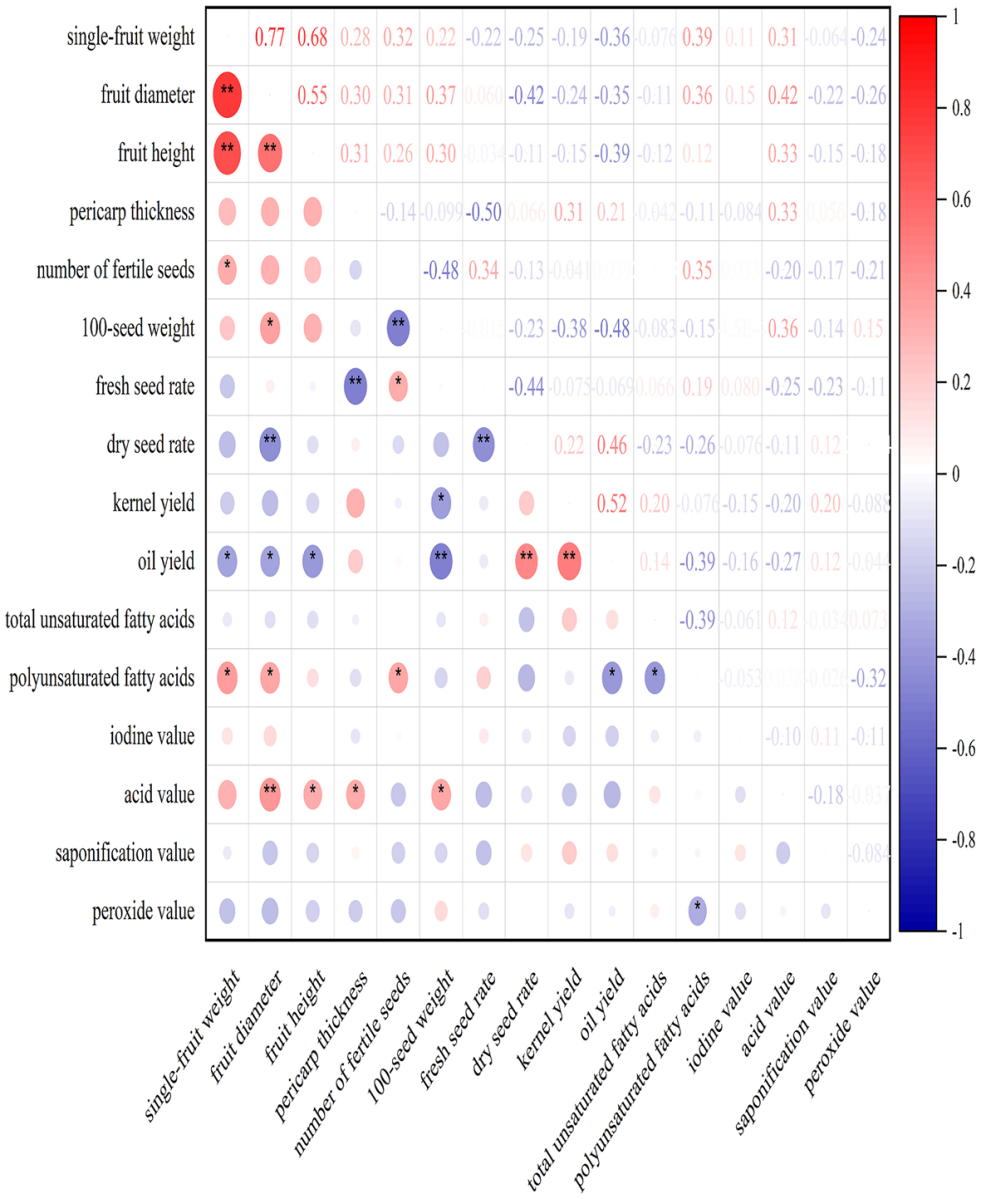
Correlation analysis of the considered characteristics.

The correlations among the characteristics are summarized in Fig. 1. Single-fruit weight had very significant correlations with the diameter and height of the fruit and significant positive correlations with the number of fertile seeds and polyunsaturated fatty acids. The number of fertile seeds exhibited a very significant negative correlation with 100-seed weight but very significant positive correlations with fresh seed rate and polyunsaturated fatty acids. 100-seed weight exhibited a very significant negative correlation with oil yield, a significant negative correlation with kernel rate and significant positive correlations with the number of fertile seeds and acid value. Fresh seed rate had a very significant negative correlation with pericarp thickness. Dry seed rate had very significant negative correlations with fruit diameter and fresh seed rate and a very significant positive correlation with oil yield. Oil yield exhibited significant negative correlations with single-fruit weight, fruit diameter and polyunsaturated fatty acids, a very significant negative correlation with 100-seed weight and very significant positive correlations with dry seed rate and kernel rate. Total unsaturated fatty acids had a significant negative correlation with peroxide value.

### Descriptive statistics and variability analysis of the seed and fruit characteristics

#### Descriptive statistical analysis

The statistical results of the 40 plants in terms of 26 indices are summarized in Table 1. The optimal values of some relatively important indices are as follows: single-fruit weight, 59.48 g; fruit diameter, 48.97 mm; fruit height, 49.59 mm; pericarp thickness, 2.61 mm; number of ventricles, 4.25; number of fertile seeds, 8.75; 100-seed weight, 684.2 g; dry seed rate, 68.8%; kernel rate, 76.5%; oil yield, 53%; oleic acid, 84.6; unsaturated fatty acids, 91.4%; polyunsaturated fatty acids, 12.2%; acid value, 0.38; iodine value, 95.9; saponification value, 204.3; and peroxide value, 0.00. Among the 40 plants, CY-6 performed the best in fruit diameter, 100-seed weight and α-linolenic acid, and CY-21 performed the best in fresh seed rate, the number of fertile seeds, stearic acid, linoleic acid, total unsaturated fatty acids and polyunsaturated fatty acids. However, the performances of these two plants in the remaining indices were common, and the optimal values of these indices were observed in different plants. These results indicate that each of the considered 40 plants owned its advantages as well as disadvantage, and therefore, it is necessary to perform comprehensive assessment to select the best germplasm resource.

**Table 1:** Descriptive statistics of the 26 indices.

	Minimum	Maximum	Average	Standard deviation	Variation coefficient (%)
Single-fruit weight (g)	22.63	59.48	35.13	7.97	22.7
Fruit diameter (mm)	36.08	48.97	41.08	2.70	6.6
Fruit height (mm)	31.86	49.59	38.38	3.43	8.9
Fruit shape index	0.92	1.28	1.08	0.08	7.6
Pericarp thickness (mm)	2.61	6.18	4.78	0.84	17.6
Number of ventricles	1.70	4.25	2.92	0.54	18.4
Number of fertile seeds	1.80	8.75	4.26	1.39	32.6
Number of abortive seeds	7.45	24.60	14.62	3.29	22.5
100-seed weight (g)	252.00	684.20	378.12	98.28	26.0
Fresh seed rate (%)	20.51	61.53	41.21	8.29	20.1
Dry seed rate (%)	34.21	68.80	57.24	6.97	12.2
Kernel rate (%)	37.35	76.47	67.93	6.64	9.8
Oil yield (%)	25.38	52.99	45.00	5.17	11.5
Iodine value (g/100 g)	71.50	95.90	82.48	5.95	7.2
Acid value (mg/g)	0.38	2.20	0.99	0.50	50.6
Saponification value (mg/g)	168.10	204.30	184.69	9.56	5.2
Peroxide value (g/100 g)	0.00	0.09	0.01	0.02	250.5
Palmitic acid (%)	6.80	9.46	8.09	0.70	8.6
Palmitoleic acid (%)	0.05	0.11	0.08	0.02	21.5
Stearic acid (%)	1.44	2.62	1.81	0.27	14.9
Oleic acid (%)	77.33	84.60	80.47	1.90	2.4
Linoleic acid (%)	5.27	11.73	8.58	1.51	17.6
*a*-linolenic acid (%)	0.25	0.71	0.38	0.09	22.9
Cis-11-eicosenoic acid (%)	0.51	0.74	0.60	0.05	8.2
Total unsaturated fatty acids	88.55	91.42	90.10	0.70	0.8
Polyunsaturated fatty acids	5.63	12.18	8.95	1.52	17.0

#### Comparative analysis of the seed and fruit economic characteristics

Of the 40 plants, the average single-fruit weight was 35.13 g, with the maximum value of 59.48 g and a maximum-minimum range of 36.85 g. The heaviest single-fruit weight was observed in CY-11, and CY-13 and CY-31 also exhibited excellent single-fruit weight, which were both higher than 50 g, whereas CY-15 had the lightest single-fruit weight (merely 22.63 g). The average pericarp thickness was 4.78 mm. CY-8 had the thinnest pericarp (2.61 mm), which was followed by CY-38 (2.68 mm), whereas CY-23, -24, and -6 had rather thick pericarps, which were all thicker than 6 mm. CY-13 exhibited the largest number of fertile sees (8.8), followed by CY-21 (7.9), and the smallest numbers were observed in CY-6 and -35 (both fewer than 2). In terms of 100-seed weight, CY-6 exhibited the best performance, which reached up to 684.2 g. This value was 107.1 g heavier than that of CY-30, which ranked the second among the 40 plants. The poorest performances were observed in CY-33 and -15, and their values were both lower than 250. The best performance in fresh seed rate was observed in CY-21 (61.53), which was followed by CY-22 (59.02), whereas CY-11 and -6 exhibited the poorest performance, whose values were both lower than 30. The average dry seed rate of the 40 plants was 57.24, and the highest was observed in CY-19, whose value reached 68.8 while the lowest was observed in CY-22 (34.21). The average kernel rate was 67.93. Both CY-25 and CY-20 had a kernel rate higher than 75, and the lowest value was observed in CY-38 (37.35). What is noteworthy is that 92.5% of the superior plants had a kernel rate higher than 60%.

#### Comparative analysis of the oil yield and fatty acids

Of the 40 plants, the average oil yield was 45%. The highest oil yield was observed in CY-37 (52.99), which was followed by CY-19 (52.25) and -22 (51.99). Among the 40 plants, only CY-30 had an oil yield lower than 30% (25.38). The average total unsaturated fatty acid content of the considered plants was 90.1. CY-10 outperformed the rest plants, whose value reached up to 91.42, whereas CY-31 exhibited the poorest performance, whose value was 88.55. The average polyunsaturated fatty acid content of the 40 plants was 8.95. CY-21, -33, -32 and -40 performed satisfactorily in this index, whose values were all higher than 11 (12.18, 11.12, 11.07 and 11.01, respectively), whereas CY-35 exhibited the poorest performance, whose value was only 5.63.

#### Comparative analysis of the oil physic-chemical characteristics

The average acid value of the seed oil samples was 0.99 mg/g, which satisfied the DB33/T 525-2004 standard of Guizhou, which stipulates that the acid value of nuisance free tea seed soil should be ≤ 1.0 mg/g. The acid values of CY-39 and -3 were rather low, which were 0.38 and 0.39, respectively. A low acid value means the existence of a small amount of free fatty acids in oil; it also means a low rancidity and a high storability of the oil. In contrast, CY-6 and -24 exhibited the highest acid value, reaching up to 2.2 mg/g. Of the 40 plants, 19 had an acid values > 0.99. Iodine value is an index that reflects the degree of unsaturation in organic compounds, a higher degree of unsaturation indicates a higher iodine value. The average iodine value of the 40 plants was 82.48. CY-29 had the highest iodine value (92.9), and CY-26, -9, -28 and -30 all had an iodine value > 90. Saponification value is an index used to test oil quality, and impure oil has a low saponification value. Among the 40 plants, CY-15 had the highest saponification value, which reached up to 204.3, and CY-18, -9 and -12 all had a saponification value above 200. Peroxide value is an index used to test oxidation degree of oil. Of the 40 plants, 29 had a peroxide value of 0, which indicated that the overall stability of *C. oleifera* in the investigated region was satisfactory.

#### Variability analysis of the characteristics

Among the 26 considered indices, peroxide value exhibited the highest variation coefficient, which reached up to 250.5%. The variation coefficient of the acid value was also high, reaching 50.6%. These results indicate that the genetic stabilities of these two plants are relatively weak, and therefore, they have great potentials in selective breeding. The variation coefficients of single-fruit weight, fertile seed number, abortive seed number, 100-seed weight, fresh seed rate, palmitoleic acid and α-linolenic acid lay between 32.6 and 20%. The variations in these indices were moderate, and therefore, these indices had certain potentials in selective breeding. The variation coefficients of the remaining indices were all lower than 20%. The variation coefficients of the fruit diameter, fruit height, fresh kernel rate, oleic acid and total unsaturated fatty acids were 6.6%, 8.9%, 9.8%, 2.4% and 0.8%, respectively, all lower than 10%, which indicate rather high genetic stabilities of these indices. The variation coefficient of total unsaturated fatty acids was the lowest, which indicates that it has the highest genetic stability.

### Comprehensive assessment

#### PCA

Sixteen out of the 26 indices were subjected to PCA, which included single-fruit weight, fruit diameter, fruit height, pericarp thickness, number of fertile sees, 100-seed weight, fresh seed rate, dry seed rate, oil yield, total unsaturated fatty acids, polyunsaturated fatty acids, iodine value, acid value, saponification value and peroxide value. The comprehensive assessment was performed as follows: (1) standardization of the original data; (2) analysis of the correlations among the characteristics; (3) extraction of the principal components with an eigenvalue > 1; and (4) construction of the comprehension function based on the contributions of the principal components. The outcomes of the PCA are summarized in Table 2. A total 6 principal components were extracted, whose cumulative contribution was 74.15%. These components basically contained the primary information of most of the original data.

**Table 2:** Coefficients, eigenvalues and contributions of the components.

	Component					
	PV1	PV2	PV3	PV4	PV5	PV6
Single-fruit weight	0.21	0.09	0.15	0.00	0.07	0.10
Fruit diameter	0.23	0.04	0.09	0.09	0.07	0.05
Fruit height	0.19	0.11	0.08	0.05	− 0.06	0.24
Pericarp thickness	0.04	0.32	0.19	0.10	0.04	− 0.06
Number of fertile seeds	0.07	− 0.22	0.28	0.13	− 0.13	0.31
100-seed weight	0.12	0.10	− 0.32	− 0.04	0.03	− 0.06
Fresh seed rate	0.02	− 0.35	− 0.02	0.19	0.02	− 0.06
Dry seed rate	− 0.13	0.17	0.09	− 0.26	− 0.25	0.37
Kernel rate	− 0.12	0.10	0.23	0.18	0.08	− 0.27
Oil yield	− 0.18	0.08	0.19	0.15	− 0.06	0.16
Total unsaturated fatty acids	− 0.05	0.01	− 0.05	0.55	0.29	− 0.03
Polyunsaturated fatty acids	0.13	− 0.17	0.18	− 0.23	− 0.17	− 0.45
Iodine value	0.04	− 0.07	− 0.01	− 0.18	0.61	0.46
Acid value	0.13	0.22	− 0.09	0.15	− 0.10	− 0.15
Saponification value	− 0.07	0.07	0.08	− 0.27	0.48	− 0.33
Peroxide value	− 0.06	0.02	− 0.26	0.06	− 0.18	0.18
Eigenvalue	3.79	2.29	2.15	1.45	1.16	1.01
Contribution (%)	23.71	14.34	13.47	9.05	7.28	6.31
Cumulative contribution (%)	23.71	38.05	51.52	60.56	67.84	74.15

As shown in Table 2, the contribution of component 1 is 23.71, and the indices with a high load included single-fruit weight (0.21), fruit diameter (0.23) and fruit height (0.19). These results indicate that the first component primarily reflects the physical characteristics of the fruit. Because single-fruit weight exhibited significant positive correlations with fruit diameter and height, it was considered as the representative factor of the first component. The contribution of component 2 is 14.34. The indices with a high positive load included pericarp thickness (0.32) and acid value (0.22), and those with a high negative load included fresh seed rate (− 0.35). Because pericarp thickness exhibited a significant positive correlation with acid value and a significant negative correlation with fresh seed rate, it was considered as the representative factor of the second component. The contribution of component 3 is 13.47. The indices with a high positive load included the number of fertile seeds (0.28) and kernel rate (0.23), and the number of fertile seeds was considered as the representative factor of the third component. The contribution of component 4 is 9.05. The index with a high positive load was total unsaturated fatty acids (the load reached up to 0.55), and therefore, it was considered as the representative factor of the fourth component. The contribution of component 5 is 7.28. The indices with a high positive load were iodine value (0.61) and saponification value (0.48). Therefore, component 5 reflects the physic-chemical characteristics of the oil. The contribution of component 6 is 6.31. The indices with a high positive load were iodine value (0.46) and dry seed rate (0.37).

#### Cluster analysis

**Figure 2:**
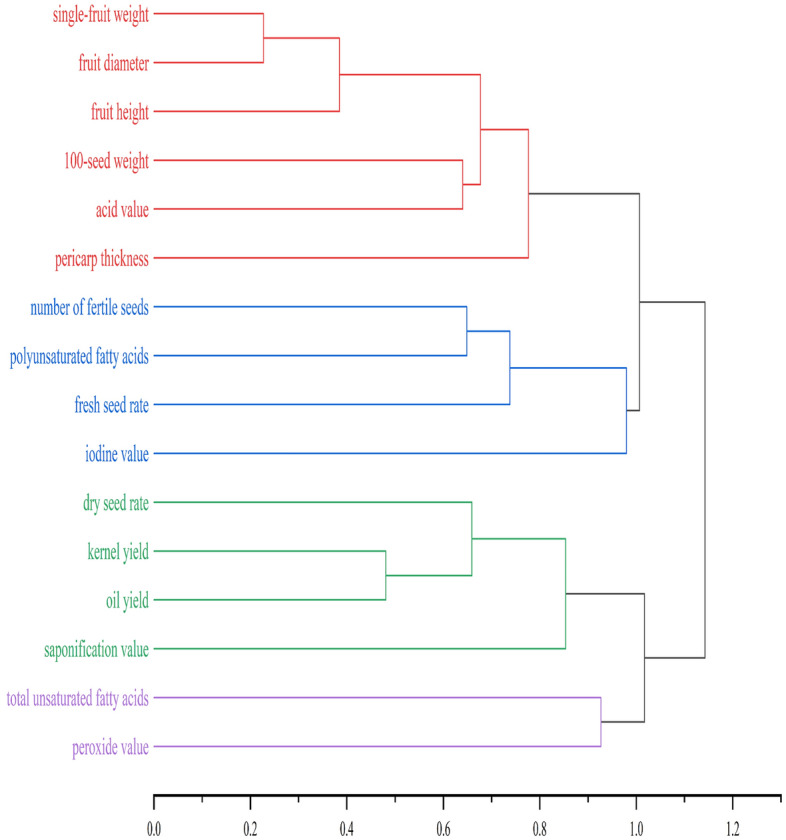
Tree structure of the cluster analysis of the 16 characteristics (drawn by Jie Qiu with Origin 2021).

Cluster analysis was conducted to group the characteristics according to the degree of similarity. The outcomes are shown in Fig. 2. At a distance of approximately 1, the 16 characteristics were clustered into four categories. The first category contained single-fruit weight, fruit diameter, fruit height, 100-seed weight, pericarp thickness and acid value, which were similar to the characteristics represented by PV1 and PV2. The second category included number of fertile seeds, fresh seed rate, iodine value and polyunsaturated fatty acids, which were similar to those represented by PV3. The third category included oil yield, dry seed rate, kernel yield and saponification value, which were similar to the characteristics represented by PV6. The fourth category consisted of total unsaturated fatty acids and peroxide value, which were similar to the characteristics represented by PV4 and PV5.

#### Ordering of the superior plants based on comprehensive assessment

The comprehensive scoring function was as follows:

$$ \begin{aligned} Zn = & {\text{ R}}_{{1}} /{74}.{15} \times {\text{PV}}_{{1}} + {\text{R}}_{{2}} /{74}.{15} \times {\text{PV}}_{{2}} + {\text{R}}_{{3}} /{74}.{15} \times {\text{PV}}_{{3}} \\ & + {\text{R}}_{{4}} /{74}.{15} \times {\text{PV}}_{{4}} + {\text{R}}_{{5}} /{74}.{15} \times {\text{PV}}_{{5}} + {\text{R}}_{{6}} /{74}.{15} \times {\text{PV}}_{{6}} \\ \end{aligned} $$

where *Z* is the comprehensive score of the plant, *n* is the designated number of the plant, R_1-6_ is the contributions of the 6 principal components, 74.15 is the cumulative contribution of the 6 principal components, and PV_1_-PV_6_ are the scores of each plant in the 6 principal components.

The principal component scores and comprehensive scores of the 40 superior *C. oleifera* trees are summarized in Table 3. The trees whose comprehensive scores among the top 10 were ordered as follows: CY-6 > CY-13 > CY-31 > CY-11 > CY-16 > CY-22 > CY-28 > CY-23 > CY-24 > CY-29. Although CY-6 exhibited poor performance in peel thickness, oil content and acid value, its performance in single fruit weight, fruit height, fruit diameter, 100-seed weight, kernel rate, polyunsaturated fatty acid content and peroxide value was excellent, thereby ranking the first. Although CY-13 exhibited poor performance in 100-seed weight, pericarp thickness and peroxide value, its performance in single-fruit weight, fruit diameter, fruit height, fertile seed number, kernel rate, oil content, total unsaturated fatty acids, acid value and saponification value was satisfactory, and therefore ranks the second.

**Table 3:** Principal component scores and comprehensive scores of the 49 superior *C. oleifera* trees.

Number	Tree designation	PV1	PV2	PV3	PV4	PV5	PV6	Comprehensive score
1	CY-6	2.81	2.06	− 1.34	− 0.05	− 0.23	− 1.61	0.89
2	CY-13	1.31	− 1.09	1.60	0.45	0.70	1.21	0.73
3	CY-31	1.95	0.55	0.59	− 1.07	− 1.12	0.79	0.66
4	CY-11	0.51	1.23	1.27	− 1.01	0.98	0.15	0.62
5	CY-16	0.98	0.18	0.57	0.25	− 0.28	0.19	0.47
6	CY-22	0.40	− 0.59	0.36	2.41	1.57	− 0.95	0.44
7	CY-28	0.72	0.28	0.32	− 0.61	0.48	1.50	0.44
8	CY-23	− 0.13	1.58	0.59	1.02	− 0.35	− 0.22	0.44
9	CY-24	0.61	1.52	0.36	1.04	− 0.59	− 2.35	0.42
10	CY-29	0.50	− 0.37	0.73	− 0.06	0.41	1.93	0.42
11	CY-5	0.16	0.63	0.85	0.65	− 1.21	1.49	0.41
12	CY-18	− 0.22	0.80	0.17	0.04	1.46	1.08	0.36
13	CY-7	− 0.08	1.30	− 0.08	0.89	− 0.32	0.56	0.34
14	CY-21	1.25	− 2.47	1.13	2.33	− 0.15	− 1.46	0.27
15	CY-27	0.29	0.09	− 0.06	− 0.10	0.34	1.20	0.23
16	CY-17	− 0.34	1.19	0.51	0.02	0.86	− 0.89	0.22
17	CY-9	− 0.56	0.96	− 0.26	− 0.25	2.37	0.40	0.19
18	CY-14	0.38	0.38	− 0.19	0.04	0.41	− 0.23	0.19
19	CY-30	2.09	− 1.13	− 1.97	− 0.60	1.24	0.45	0.18
20	CY-40	0.38	− 0.25	0.92	− 0.51	− 1.53	− 0.02	0.03
21	CY-36	0.36	0.44	0.30	− 1.24	− 0.59	− 1.13	− 0.05
22	CY-10	− 1.12	0.00	− 0.53	1.01	1.02	1.13	− 0.13
23	CY-26	− 0.40	− 0.99	0.57	− 0.56	0.75	0.72	− 0.15
24	CY-2	− 0.61	− 0.19	0.01	0.64	− 0.89	0.89	− 0.17
25	CY-25	− 0.77	− 0.13	− 0.08	2.04	− 1.15	− 0.59	− 0.20
26	CY-3	− 0.57	0.34	− 0.19	− 0.71	0.18	− 0.62	− 0.27
27	CY-19	− 1.51	0.57	0.44	0.02	− 0.42	0.67	− 0.28
28	CY-34	− 1.41	− 0.19	− 0.02	0.90	0.46	0.01	− 0.33
29	CY-1	− 1.01	− 0.52	0.20	0.43	0.16	− 0.20	− 0.34
30	CY-37	− 0.64	− 0.09	0.75	− 1.32	− 1.38	− 0.01	− 0.38
31	CY-33	− 0.04	− 1.51	0.78	− 0.83	− 1.08	− 0.29	− 0.40
32	CY-4	0.08	− 0.42	− 1.81	− 1.14	1.46	− 0.63	− 0.44
33	CY-39	− 0.98	− 0.13	0.54	− 1.06	− 0.96	0.19	− 0.45
34	CY-12	− 0.87	0.06	− 0.65	− 0.80	0.68	− 1.09	− 0.51
35	CY-32	− 0.42	− 0.74	0.62	− 1.24	− 1.11	− 1.27	− 0.53
36	CY-20	− 1.05	− 0.74	− 0.25	0.20	0.46	− 0.95	− 0.53
37	CY-38	1.01	− 1.74	− 2.22	− 0.43	− 1.28	0.63	− 0.54
38	CY-35	− 1.18	1.36	− 3.06	0.96	− 1.96	1.07	− 0.66
39	CY-8	− 0.40	− 1.74	− 1.74	0.23	− 0.14	− 0.17	− 0.78
40	CY-15	− 1.45	− 0.48	0.28	− 1.94	0.76	− 1.58	− 0.80

The highest score in the first principal component was observed in CY-6, which indicated that CY-6 had the best performance in single-fruit weight, fruit diameter, fruit height, 100-seed weight, polyunsaturated fatty acids and fatty acids. High scores in the second principal component were observed in CY-6, -23 and -24. High scores in the third principal component were observed in CY-13 and -11, which indicated that these two plants performed well in the number of fertile seeds and kernel rate. According to the results of this study, CY-13 ranked the first in the number of fertile seeds; it also had a satisfactory kernel rate. High scores in the fourth principal component were observed in CY-22 and -21, which indicated that these two plants performed well in total unsaturated fatty acids. This result was supported by the actual determination in this study, according to which the total unsaturated fatty acid content of CY-21 ranked the first and that of CY-20 was close to the maximum. The highest score in the fifth principal component was observed in CY-9, which indicated that CY-9 had the highest iodine and saponification values. This result was consistent with the values actually determined in this study: Both the iodine value and the saponification value were close to the maximum values among the investigated plants. High scores in the sixth principal component were observed in CY-29, -28 and -5, which indicated that these three plants had excellent performance in iodine value and dry kernel rate. The actual measurements showed that the CY-29 had the highest iodine value and CY-29 had an iodine value close to the maximum. In addition, the dry kernel rates of both plants were close to the maximum. Therefore, the outcomes of PCA were basically consistent to those actually determined in this study, which indicates the feasibility of the assessment system used in this study.

Table 4 shows the average yields per unit crown width of the 40 trees in five years. As shown in the table, CY-6 and CY-13 with the top two yields were also the two plants ranking the first and second according to comprehensive evaluation. In addition, among the top 10 plants in terms of comprehensive ranking, seven had a yield per unit crown width ranked among the top 10.

**Table 4:** Average yields per unit crown width of the 40 plants in five years.

Serial no	Average yield (kg/m^2^)	Comprehensive ranking	Yield ranking
CY-6	5.86	1	1
CY-13	5.743	2	2
CY-22	5.702	6	3
CY-31	5.431	3	4
CY-7	5.411	13	5
CY-16	5.266	5	6
CY-21	5.121	14	7
CY-18	5.098	12	8
CY-11	5.031	4	9
CY-23	4.913	8	10
CY-24	4.684	9	11
CY-29	4.659	10	12
CY-5	4.389	11	13
CY-28	4.228	7	14
CY-17	4.197	16	15
CY-27	4.077	15	16
CY-30	4.040	19	17
CY-2	4.020	24	18
CY-14	3.544	18	19
CY-36	3.526	21	20
CY-9	3.416	17	21
CY-40	3.320	20	22
CY-25	3.315	25	23
CY-19	3.059	27	24
CY-26	3.016	23	25
CY-1	2.946	29	26
CY-10	2.930	22	27
CY-34	2.914	28	28
CY-35	2.935	38	29
CY-8	2.795	39	30
CY-37	2.787	30	31
CY-38	2.768	37	32
CY-33	2.653	31	33
CY-12	2.452	34	34
CY-32	2.452	35	35
CY-15	2.415	40	36
CY-4	2.225	32	37
CY-20	2.090	36	38
CY-3	2.010	26	39
CY-39	1.999	33	40

## Discussion

Comparison between the data obtained in this study and those in studies on other *C. oleifera* cultivars shows that *C. oleifera* grown in the investigated region exhibited great advantages in single-fruit weight, total unsaturated fatty acid content and kernel yield. The unsaturated fatty acid of *C reticulate* Lind1 f. grown in Tengchong, Yunan Province, is 82.07%^28^. *Camellia oleifera* of “Xianglin” series grown in Guangxi Province has a single-fruit weight of 23.53 g, a kernel yield of 57.5% and a total unsaturated fatty acid content of 89.9%^29^. *Camellia oleifera* of “Changlin” series grown in Zhejiang Province has a single-fruit weight of 18.92 g^30^. The 18 superior trees of the hybrid F1 generation of “Youza 2” and “Huashuo” has a fruit-single weight of 23.66 g and a total unsaturated fatty acid content of 89.1%^31^. The average kernel yield and total unsaturated fatty acid content of 30 superior trees from six main production areas of *C. oleifera* are 60.5% and 88.8%, respectively^32^. In this study, *C. oleifera* grown in the low-hot valley area had a single-fruit weight of 35.13 g, a total unsaturated fatty acid content of 90% and a kernel yield of 67.9%, showing great advantages over other cultivars reported in literature. However, *C. oleifera* grown in the low-hot valley area did not show a satisfactory fresh seed rate. The reasons for these differences may be that the unique climate in low-hot valleys benefits organic matter accumulation while the light, temperature and water conditions during the differentiation of flower buds are harmful to the differentiation of flower buds of *C. oleifera*. Total unsaturated fatty acids are the collective term of oleic acid, linoleic acid, linolenic acid, palmitoleic acid and cis-11-eicosenoic acid. The formation mechanism underlying the formation of unsaturated fatty acids in higher plants is as follows: Stearoyl carrier protein desaturase (SAD) catalyzes the desaturation of stearic acid to generate oleic acid, which is regulated by temperature, darkness and injury^33,34^; the activity of SAD does not only significantly affects the ratio of saturated fatty acids to unsaturated fatty acids but significantly enhances the resistance of plants to low temperature as well^35,36^. Therefore, it is reasonable to presume that during the oil accumulation period of *C. oleifera* in the investigated region, SAD activity is high, which is beneficial for the first-step reaction, i.e., the desaturation of stearic acid; under the action of oleate dehydrogenase (Δ12FAD or FAD2), polyunsaturated fatty acids further saturate the generated linoleic acid and α-linolenic acid while oleic dehydrogenase controls the contents of oleic acid and linoleic acid as well as their ratio (O/L)^37,38^ (however, we did not include the O/L ratio as an index). In a previous study, our team compared the fatty acid composition of *C. oleifera* with those of other oil crops, and the results showed that the content of mono-unsaturated fatty acids in *C. oleifera* oil was much higher than those in the vegetable oil that is commonly available in market, such as rapeseed oil, soybean oil, peanut oil, sunflower oil and olive oil. This result indicates that during the oil formation process of *C. oleifera*, SAD activity is high but FAD2 activity is low, which cause the oleic acid content to be maintained at a high level (as a consequence, oleic acid does not continues to, or only a small amount of oleic acid, transform to other unsaturated fatty acids); therefore, in *C. oleifera* oil, the contents of unsaturated fatty acids, except for that of oleic acid, are all low. Nowadays, olive oil is popular among consumers because its linolenic acid/linoleic acid ratio is generally higher than those in other oil substances, and the ideal standard for the linolenic acid/linoleic acid ratio in the best edible vegetable oil is 1:1. Because the linolenic acid/linoleic acid ratios in soybean oil, rapeseed oil and other oils consumed by Chinese people for a long time are far lower than those in olive oil and tea oil, a large amount of linoleic acid accumulate in the body; therefore, more linolenic acid to neutralize or less linoleic acid should be taken in^39^. In this study, oleic acid had a negative correlation with linoleic acid and a very significant positive correlation with the ratio between linolenic acid and linoleic acid. The high oleic acid content in tea oil indicates that its linoleic acid content is far lower than those in other vegetable oils. Therefore, tea oil may be more beneficial for Chinese people’s health than olive oil.

In this study, the results of the comprehensive assessment were basically consistent with the yields per unit crown width. However, there were differences. For instances, some plants, such as CY-7, CY-21 and CY-18, had high yields but were relatively low-ranked down the scale based on comprehensive assessment, whereas the plants CY-11, CY-28, CY-9 and CY-3 exhibited the opposite. These differences further indicate that the assessment for *C. oleifera* should not be confined to the single index yield. PCA is an analytical method in which multiple variables are integrated into a small number of variables without losing information of multiple related characteristics. Wang et al.^29^ used this method to analyze the 13 characteristics of “Xianglin” *C. oleifera* and extracted three principal components. According to them, the indices with high loads of the three principal components included fruit height, single-fruit weight, dry oil yield, 100-seed weight, fruit shape index and fresh oil yield. In a previous study, our team used PCA to analyze the 16 characteristics of *Camellia weiningensis* Y.K.Li.sP.nov. and extracted three principal components. The indices with high loads of the three principal components included oleic acid, fruit diameter, single-fruit weight, fruit shape index, pericarp thickness and dry kernel rate. In this study, the indices with high loads of 6 principal components consisted of single-fruit weight, pericarp thickness, fertile seed number, dry kernel rate, total unsaturated fatty acids, iodine value, dry seed rate, and so on. Although the characteristic indices for PCA vary according to *C. oleifera* cultivars, the final extracted principal components all include the fruit number characteristics, kernel economic characteristics and oil characteristics, which indicate that for the purpose of *C. oleifera* assessment, comprehensive factors should be considered. In the meantime, although the comprehensive evaluation systems of different *C. oleifera* cultivars are similar, there are differences. Therefore, in the comprehensive evaluation of the economic characteristics of *C. oleifera*, it is necessary to select the comprehensive evaluation indices according to different needs.

In this study, the oil yield of *C. oleifera* grown in the low-hot valley area exhibited a very significant positive correlation with dry seed rate (r = 0.462). This results is consistent with the result based on 1361 different *C. oleifera* germplasms (r = 0.33) reported by Chen et al.^40^. Pericarp thickness exhibited a very significant negative correlation with fresh seed rate. According to the study conducted by Zhu and Shi^28^, pericarp thickness is negatively correlated with seed yield, and the seed yield decreases by 27% when the pericarp thickness increases by 1 cm. Our result is basically consistent with Zhu and Shi’s^28^. However, our study showed that in *C. oleifera* grown in the low-hot valley area, the dry seed rate exhibited a very significant negative correlation with the fresh seed rate (r = 0.439), which was quite different from those reported in the literature. For instances, in *C. oleifera* grown in Hu’an County, Hubei Province, the fresh seed rate is positively correlated with the dry seed rate (r = 0.32)^41^; based on the analysis of 1361 different *C. oleifera* germplasms, fresh seed rate has a very significant positive correlation with dry seed rate (r = 0.74), and the fresh seed rate in *C. meiocarpa* exhibits a very significant positive correlation with the dry seed rate (r = 0.79)^42^. Presumably, the reasons for these differences are as follows. The main difference between dry seeds and fresh seeds lies in water content. Fresh seed rate is an unstable index, and the water content of fresh seeds of *C. oleifera* under different climate varies greatly. Furthermore, the effect of production sites on dry seed rate and fresh seed rate are also significant. In addition, the fruit-picking time can also significantly affect the water content of fresh seeds^43^, which further affects the correlation between dry seed yield and fresh seed yield. However, these presumptions remain to be validated in the future. In addition, as this study was mainly to select the cultivars with satisfactory economic fruit characteristics, research flowers, leaves and tree growth has not been conducted yet at the current stage. The research in these aspects will undoubtedly deepen the systematic understanding of *Camellia oleifera* in the low heat river valley and provide useful data for the promotion of the species in areas with similar environmental conditions in the world.

## Materials and methods

### Experimental site

**Figure 3:**
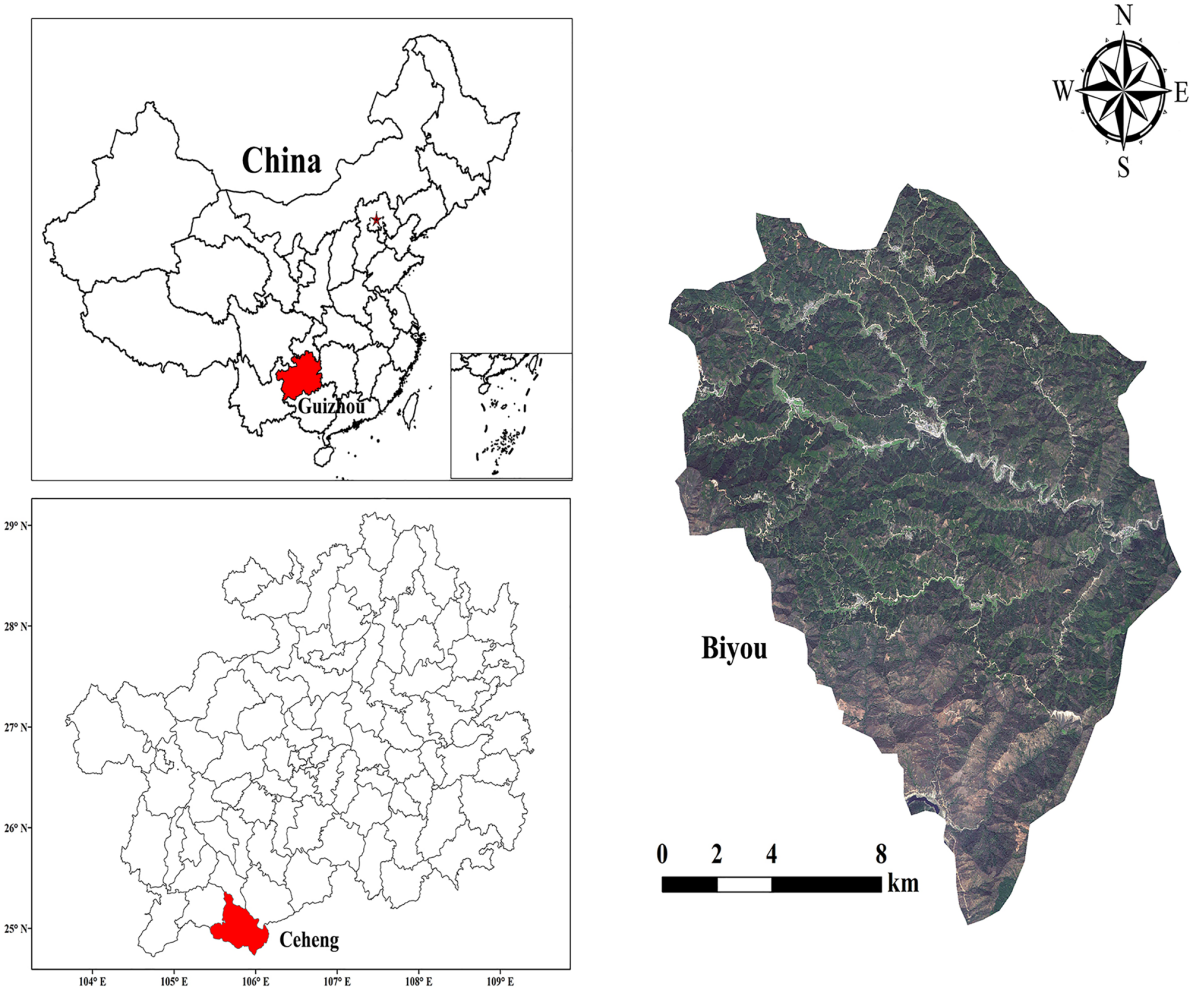
Satellite maps of Biyou Town, Ceheng County, China (created with GGGIS Map Downloader, V1.02, http://gggis.com/).

The experimental site is located at Ceheng County, Qianxinan Buyi and Miao Autonomous Prefecture, Southwest Guizhou Province, China (24.71°–24.94° N and 105.79°–106.05° E; Fig. 3). It is at the intersection of Nanpan River and Beipan River, two tributaries of the upper reaches of the Pearl River, and its terrain belongs to typical low-hot valleys. The climate of Cecheng County is subject to subtropical warm humid monsoon climate. In this region, there is no severe cold weather during the flowering period of *C. oleifera*. There, the average annual sunshine hours is 1514 h, the average annual temperature is 19.2 °C, with the extreme minimum temperature of − 4 °C, the average frost-free period is 345 d and the average annual rainfall is 1340.7 mm. At the experimental site, the soil is slightly acidic.

### Experimental materials

All experimental *C. oleifera* plants in this study were from the *Camellia oleifera* Germplasm Resources Nursery, located at Biyou Town, Ceheng County (Table 5). Prior to the establishment of the nursery, the *C. oleifera* research team of Guizhou University conducted an investigation of *C. oleifera* resources in Ceheng County. The germplasm resources with excellent performances in yield (high and stable) and disease resistance were selected and their seeds were collected. The seeds were sown at the nursery in 2007, with a density of 2000 per hectare, and three repetitions were set for each resource.

**Table 5:** Source of the 40 *C. oleifera* plants.

Serial no	Germplasm type	Germplasm source	Planting site	Planting year	Plant age (year)
CY-1-CY-4	Wild seedling	Badu Town	Biyou nursery	2007	14
CY-5- CY-10	Wild seedling	Yata Town	Biyou nursery	2007	14
CY-11-CY-15	Wild seedling	Qiaoma Town	Biyou nursery	2007	14
CY-16-CY-19	Wild seedling	Qiaoma Town	Biyou nursery	2007	14
CY-20-CY-26	Wild seedling	Biyou Town	Biyou nursery	2007	14
CY-27-CY-31	Wild seedling	Biyou Town	Biyou nursery	2007	14
CY-32-CY-37	Wild seedling	Biyou Town	Biyou nursery	2007	14
CY-38-CY-40	Wild seedling	Biyou Town	Biyou nursery	2007	14

**Figure 4:**
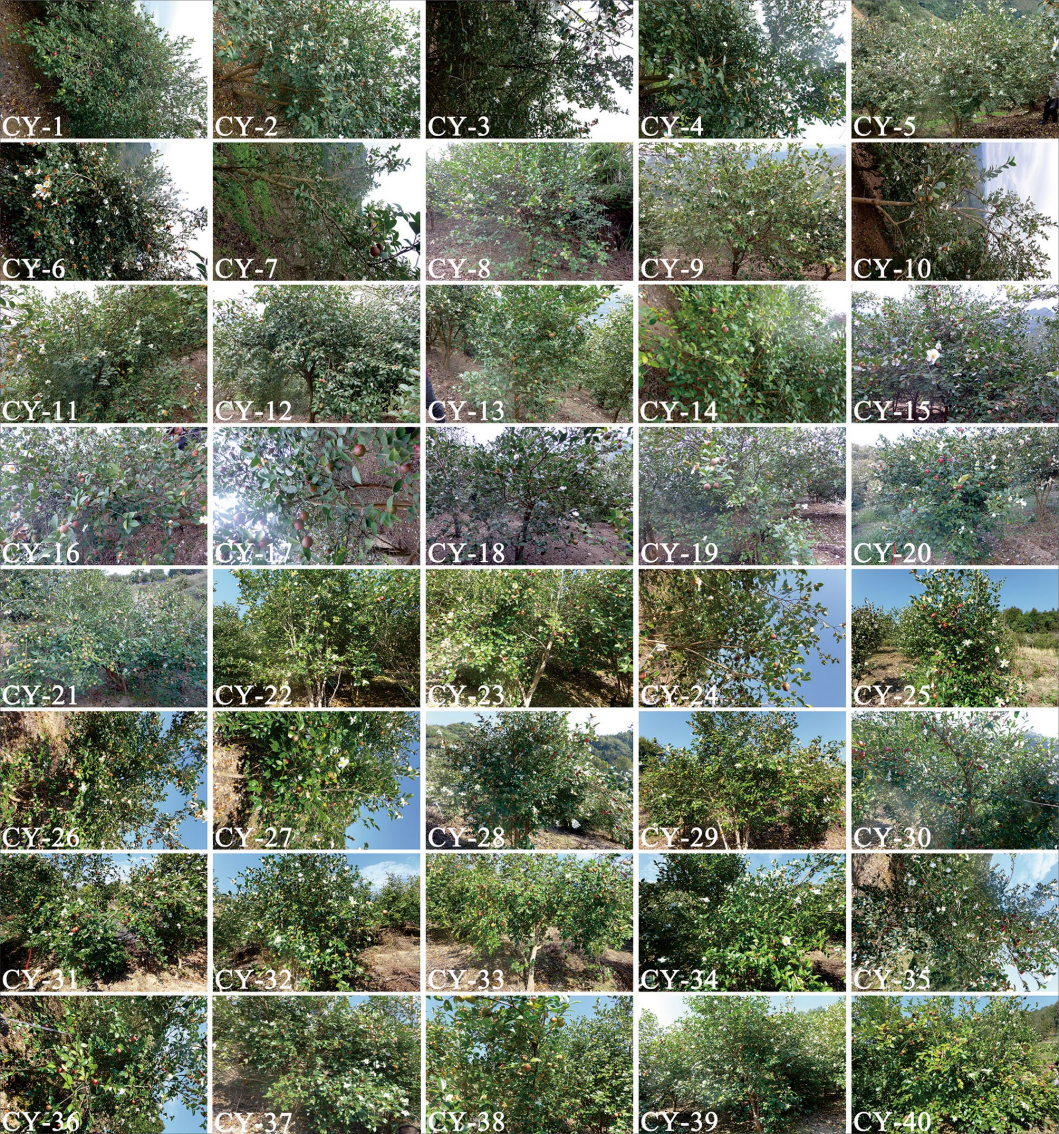
Forty superior *C. oleifera* plants.

**Figure 5:**
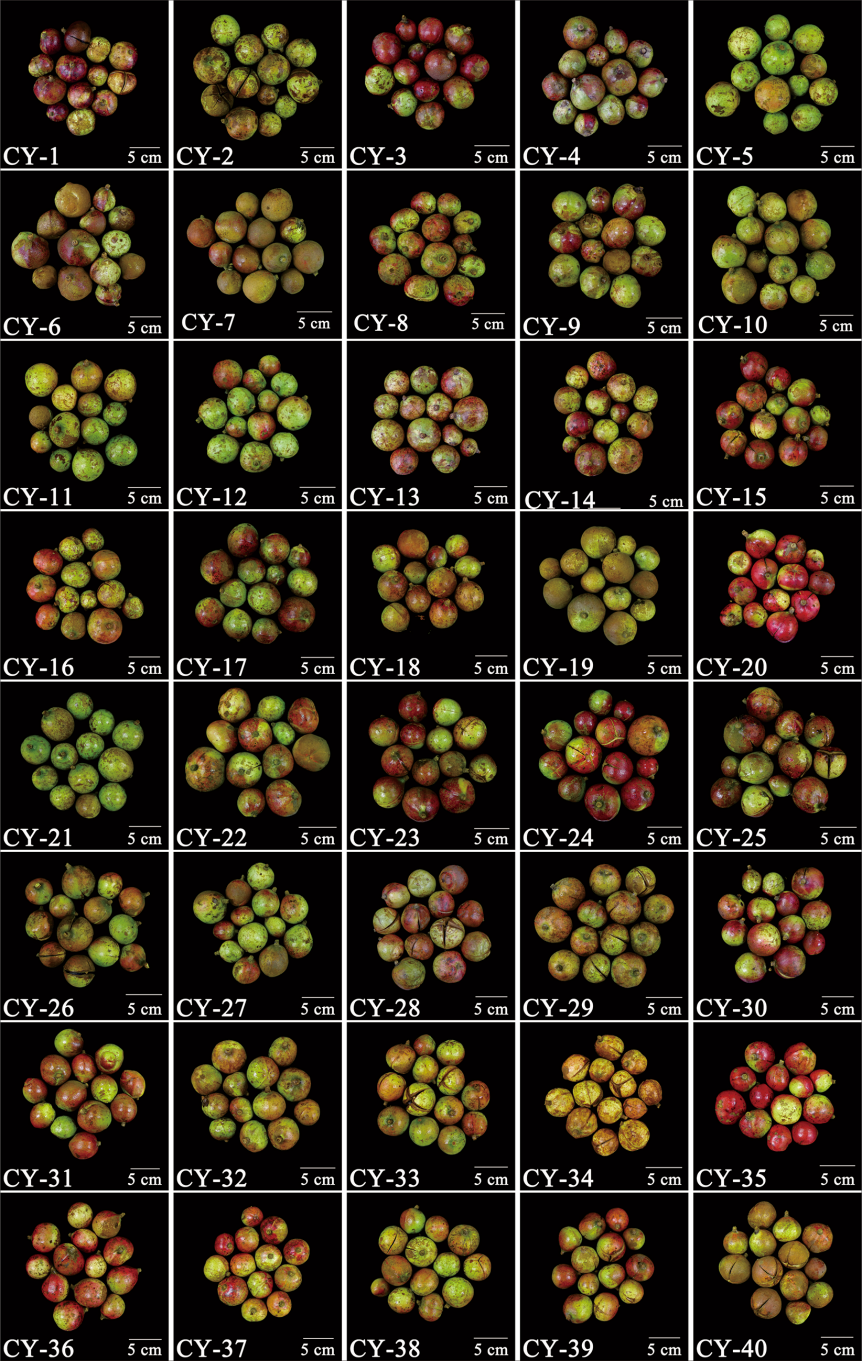
Mature fruits of 40 superior *C. oleifera* plants.

At the nursery, the soil was typical yellow soil, which was located on the sunny south slope of the same plot. All plants were watered once per month and fertilized once per year (organic fertilizer at 5 kg after the plant grew up). The trees grew naturally. Based on five consecutive years’ observations, 40 excellent plants were randomly selected, which had healthy growth, a high seed-setting rate after natural pollination, a stable yield and were free from diseases or insect pests (Fig. 4). These trees were randomly designated as “CY-(code number)”. In the full-fruit season, 30 mature fruits (identified based on slight cracking of the pericarp) were randomly sampled from each plant (Fig. 5), and they were bagged and labeled for later use.

All experiments involved in this study followed relevant local guidelines and gained permissions from Ceheng Forestry Bureau.

### Determination of economic characteristics

#### Phenotypic characteristics

The sample fruits in each bag were well mixed, and 5 were randomly selected. The single-fruit weight, fruit diameter, fruit height and pericarp thickness were measured with an electronic digital caliper (Mitutoyo 500-197-20; Japan; precision, 0.01 mm), and the 100-seed weight, fresh seed weight, dry seed weight and dry kernel weight were determined with an electronic balance (CP522C; precision, 0.01 g). In addition, the numbers of ventricles, fertile seeds and abortive seed in the fruit were counted. For fruit diameter, two sides of the fruit were measured, and for pericarp thickness, four points were measured for each fruit. For the remaining characteristics, measurement was performed once. The averages of the fruit shape index, fresh seed rate, dry seed rate and kernel rate were obtained based on the following formula:

$$ \begin{gathered} {\text{Fruit shape index }} = {\text{ fruit diameter}}/{\text{fruit height}}; \hfill \\ {\text{Fresh seed rate }} = {\text{ fresh seed weight}}/{\text{fruit weight}} \times {1}00\% ; \hfill \\ {\text{Dry seed rate }} = {\text{ dry seed weight}}/{\text{fresh seed weight }} \times {1}00\% ; \hfill \\ {\text{Kernel rate }} = {\text{ dry kernel weight}}/{\text{dry seed weight }} \times {1}00\% \hfill \\ \end{gathered} $$

#### Determination of fatty acid related indices

1. Oil yield. Determination was performed using the Soxhlet extractor method, and the specific procedures were as follows. Kernels were dried at 80 °C for 24 h and then ground. Approximately 10 g of the sample was wrapped in dried 12 cm filter paper (weight, W_0_) and then dried to constant weight. After cooling, the filter paper and the sample were accurately weighed (total weight, W_1_). The filter paper package with the sample was placed into a Soxhlet extractor for 10-h circular extraction with petroleum ether. The package was then removed, dried at 105 °C and then weighed (total weight of the filter paper and residue, W_2_).
  $$ {\text{Oil yield }} = \, \left( {{\text{W}}_{{1}} - {\text{W}}_{{2}} } \right)/\left( {{\text{W}}_{{1}} - {\text{W}}_{0} } \right) \times { 1}00\% $$
2. Iodine value. Specifically, approximately 200 g of the oil sample were weighed (precision, 0.001 g) into a 500-ml conical bottle. Afterwards, 20 ml of the mixture of cyclohexane and glacial acetic acid according to a volume ratio of 1:1 was added. After well mixed, 25 ml of Wijs reagent was added. The solution was let stand in the dark for 1 h. Potassium iodide solution at 20 ml and purified water at 150 ml were separately added. Labeled sodium thiosulfate standard solution was dripped into the sample solution till the yellow color of iodine almost disappeared. Starch solution was dripped, during which the bottle was shaken vigorously until the blue color disappeared. Blank tests and control tests were performed simultaneously. The iodine value was calculated based on the following formula:
  $$ I_{v} = \left[ {{12}.{69}*C\left( {V_{1} - V_{2} } \right)} \right]/m $$
  where *I*_*v*_ is the iodine value of the sample (g/100 g), *C* is the concentration of the sodium thiosulfate solution (mol/L), *V*_*1*_ is the volume of the sodium thiosulfate solution consumed by the blank solution (mL), *V*_*2*_ is the volume of the sodium thiosulfate solution consumed by the sample solution (mL), and *m* is the mass of the sample (g).
3. Acid value. The acid value was determined using the cold solvent indicator titration method. Specifically, approximately 20 g of the oil sample (prevision, 0.05 g) were weighed into a 250-ml conical bottle. Ether-isopropanol solution at 50 mL and 3–4 drops of phenolphthalein (indicator) were added. The solution was well shaken, and standard titration solution of sodium hydroxide was then applied. When the sample solution appeared slightly red and this color did not fade within 15 s, titration was terminated. Blank tests and control tests were performed simultaneously. The acid value was calculated based on the following formula:
  $$ W_{1} = \left[ {{56}.{1}*C\left( {V_{1} - V_{0} } \right)} \right]/m $$
  where *W*_*1*_ is the acid value of the sample (mg/g), *C* is the concentration of the standard sodium hydroxide titration solution (mol/L), *V*_*1*_ is the volume of the sodium hydroxide solution consumed by the sample solution (mL), *V*_*0*_ is the volume of the sodium hydroxide solution consumed by the blank solution (mL), and *m* is the mass of the sample (g).
4. Saponification value. Approximately 2 g of the oil sample (prevision, 0.005 g) were weighed into a 250-ml alkali-resistant conical bottle. Potassium hydroxide-ethanol solution at 25 mL and a small amount of zeolite were added. The oil mixture was kept boiled for 60 min on a condensation reflex device. A few drops of phenolphthalein were applied. Hydrochloric acid standard solution at 0.5 mol/L was used for titration, which was terminated when the pink color of the sample solution disappeared. Blank tests and control tests were performed simultaneously. The saponification value was calculated based on the following formula:
  $$ W_{2} = \left[ {{56}.{1}*C\left( {V_{1} - V_{0} } \right)} \right]/m $$
  where *W*_*2*_ is the saponification value of the sample (mg/g), *C* is the concentration of the hydrochloric acid standard solution (mol/L), *V*_*1*_ is the volume of the hydrochloric acid solution consumed by the sample solution (mL), *V*_*0*_ is the volume of the hydrochloric acid solution consumed by the blank solution (mL), and *m* is the mass of the sample (g).
5. Peroxide value. Approximately 3 g of the oil sample (prevision, 0.001 g) were weighed into a 250-ml iodine flask. Chloroform-ice ethanol mixture at 30 mL was added, and the mixture was softly shaken till the sample solution was completely solved. Saturated potassium iodide solution at 1.00 mL was added. After softly shaken for 30 s, the sample solution was let stand in the dark for 3 min. Purified water at 100 mL was added. Sodium thiosulfate standard solution was used for titration till the sample solution appeared light yellow. The sample solution was titrated with 1 mL of starch solution till the blue color disappeared. Blank tests and control tests were performed simultaneously. The peroxide value was calculated based on the following formula:
  $$ W_{3} = \left[ {{1}00*C\left( {{\text{V}}_{{1}} - {\text{ V}}_{0} } \right)*0.{1269}} \right]/m $$
  where *W*_*3*_ is the peroxide value of the sample (mg/g), *C* is the concentration of the sodium thiosulfate standard solution (mol/L), *V*_*1*_ is the volume of the sodium thiosulfate solution consumed by the sample solution (mL), *V*_*0*_ is the volume of the sodium thiosulfate solution consumed by the blank solution (mL), and *m* is the mass of the sample (g).
6. Fatty acid composition and content determination. Fatty acid composition and content determination was performed in accordance with the method described in the literature^44^. The components of fatty acids were determined using the basic methyl esterization method. The oil sample was reacted with methanol to prepare fatty acid methyl ester, which was followed by gas chromatography. Specifically, 4 g of the oil sample was added into a round-bottom flask. Methanol (40 mL) and potassium hydroxide-methanol solution (0.5 mol/L) at 2 mL were added. The reflux device was connected, and the sample solution was heated and refluxed till becoming clear and apparent. After the flask was cooled, the liquid in it was transferred into a separating funnel. The flask was rinsed with 20 mL of n-heptaine, and the rinsing liquid was also poured into the separating funnel. Distilled water of 40 mL was added into the funnel. The solution was shaken evenly and then let stand for layer separation (the upper layer was the lipid layer and the lower was the water layer). Extraction was continued with 20 mL of n-heptaine, and the extracted upper-layer solution was merged with the lipid layer. The obtained n-heptaine solution containing fatty acid esters was rinsed several times until the waste water became neutral. The lipid layer was isolated. The lipid-layer solution was dried with anhydrous sodium sulfate, which then underwent filtration and evaporation. n-heptane solution containing fatty acid methyl esters (about 20 mL) was obtained for a later use.

The conditions of gas chromatography were as follows: detector, FID; column type and specification, SP2340; chromatographic column, 60 m*0.25 111/11 × 0.2 μm; temperature program, initial temperature (50 °C, 2 min) to 170 °C (10 °C/min; 10 min), 180 °C (2 °C/min; 10 min) and then 220 °C (4 °C/min; 22 min); Inlet temperature, 250 °C; detector temperature, 300 °C; carrier gas, nitrogen; split ratio, 1:50; sample size, 1 μL.

The components of fatty acids were determined based on comparisons with the retention times of samples of different fatty acid standards. The relative content of each component was calculated using the area normalization method. The experiment was repeated three times, and an average value of each component was obtained^45^.

### Determination of the yield per unit crown width

Yield per unit crown width also constitutes an important index in excellent germplasm screening. In actual practice, it is unreasonable to consider fruit characters only while ignoring the yield. To further improve the comprehensive evaluation system of the 40 excellent *C. oleifera* in the low heat valley of Guizhou, the five-year yields per unit crown width of the plants were also taken into account in this study. In full fruit period, the crown width of each plant was measured using the crown projection method: The projected areas of the crown in the east–west and north–south directions were measured, respectively. Specifically, when the projection was close to elliptical, the long axis of the ellipse was marked as *a* and the short axis was marked as *b*, and the crown area was calculated based on the formula *S* = Π*ab*; when the projection was close to circular, the radius *r* was measured, and the crown area was calculated based on the formula *S* = Π*r*^2^. All fruit was collected and weighed (accurate to 0.1 kg). The yield per unit crown width was calculated as follows:

Yield per unit crown width = total yield/crown area.

### Statistical analysis

Software for the analysis and processing of the data and graphs included WPS Office 2019, PS 2020 and SPSS25. The correlations among the characteristics were tested with Kaiser–Meyer–Olkin (KMO) and Bartlett sphericity and analyzed with the Pearson method. PCA was performed through dimension reduction.
